# Radioiodine treatment in female survivors of pediatric differentiated thyroid carcinoma does not affect future pregnancy rates

**DOI:** 10.20945/2359-4292-2023-0505

**Published:** 2024-10-04

**Authors:** Marise Codeco de Andrade Barreto, Natalia Treistman, Lara Bessa Campelo Pinheiro Cavalcante, Daniel Bulzico, Fernanda A. de Andrade, Rossana Corbo, Paulo Alonso Garcia Alves, Fernanda Vaisman

**Affiliations:** 1 Instituto Nacional de Câncer Departamento de Endocrinologia Oncológica Rio de Janeiro RJ Brasil Departamento de Endocrinologia Oncológica, Instituto Nacional de Câncer (Inca), Rio de Janeiro, RJ, Brasil; 2 Universidade Federal do Rio de Janeiro Faculdade de Medicina Departamento de Endocrinologia Rio de Janeiro RJ Brasil Departamento de Endocrinologia, Faculdade de Medicina, Universidade Federal do Rio de Janeiro (UFRJ), Rio de Janeiro, RJ, Brasil

**Keywords:** Pregnancy, radioiodine, pediatric thyroid cancer

## Abstract

**Objective:**

Patients with pediatric differentiated thyroid carcinoma (DTC) treated with radioiodine (RAI) therapy may experience long-term side effects, such as gonadal dysfunction. Therefore, it is crucial to understand the impact of this therapy on ovarian reserve and future pregnancy rates.

**Subjects and methods:**

Retrospective analysis of 64 female DTC survivors of childbearing age to assess the risk of infertility due to RAI performed before the age of 19 years.

**Results:**

Thirty-two out of the 64 DTC survivors had a history of at least one pregnancy during follow-up. No significant differences were observed between the cumulative RAI activity, treatment regimens (multiple versus single RAI treatment), age at first treatment, or presence of lymph node or distant metastases. Notably, the group without a history of pregnancy had a younger age at the time of diagnosis and larger tumors. Age at first pregnancy was slightly higher than that in the general population, but no increase in negative maternal or fetal outcomes was observed.

**Conclusions:**

The results of this study show little observational evidence suggesting important adverse effects of RAI on fertility or pregnancy outcomes among female survivors of childhood DTC. Still, studies including larger populations are warranted.

## INTRODUCTION

Childhood cancer survivors (CCSs) have been increasing in number and represent a population with a substantial risk of long-term complications due to their cancer treatments (1,2). Depending on the type of cancer therapy, the incidence of treatment consequences in the long term may vary. An important late treatment complication among CCSs is gonadal dysfunction (1).

Multiple studies (3,4) have highlighted the wide-ranging effects of chemotherapy and radiotherapy on gonadal function. The risk of premature ovarian insufficiency (POI) and/or profound diminished ovarian reserve with resulting fertility impairment is a potential effect being studied in patients following radioiodine (RAI) therapy for differentiated thyroid carcinoma (DTC).

Studies on the risks of unfavorable reproductive outcomes after RAI therapy – such as miscarriage, impaired fertility, preterm delivery, or genetic damage leading to congenital malformations and malignancies – are debatable (5). Two recent meta-analyses (6,7) concluded that poor pregnancy outcomes, including miscarriage, preterm labor, and congenital anomalies, are not a long-term effect of RAI. However, only a few studies have focused solely on CCSs of DTC (8,9).

Nies and cols. (8) and Albano and cols. (9) found no association between RAI doses and fertility problems in, respectively, 56 and 72 female CCSs of DTC. However, some concern remains about the effect of RAI cumulative doses and long-term outcomes among these patients, especially in CCSs who were treated during the pubertal maturation phase, and whether this treatment could decrease fertility in the adult phase. Our group recently showed the impact of multiple RAI treatments, regardless of cumulative activity, on serum anti-Müllerian hormone (AMH) levels in CCSs who underwent RAI treatment during childhood, indicating that multiple ovarian injuries may affect future follicular reserve (10). The aim of the present study was to assess whether RAI therapy reduces pregnancy rates among female survivors of childhood DTC.

## SUBJECTS AND METHODS

**Figure 1:**
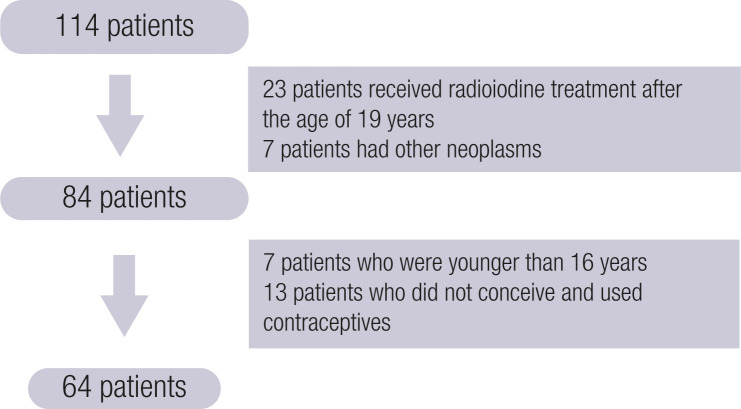
Flowchart of the study cohort for analysis of female fertility.

A cohort of 114 female patients who were younger than 19 years at diagnosis was treated at the National Cancer Institute (INCA) and at the Federal University of Rio de Janeiro (UFRJ) between 1980 and 2023. Twenty-three patients treated with RAI therapy after the age of 19 years and 7 patients with other neoplasms before or after DTC were excluded. We further excluded patients younger than 16 years and those who did not conceive, used contraceptives at the time of the study, and had never tried to become pregnant before (Figure 1).

Patients with at least 6 months of follow-up post-RAI were included. Permission was requested, and informed consent for participation was obtained from all participants. The protocol of the study was approved by CEP/CAAE (66569517.8.0000.5257). The study involved the collection of data from physical and electronic medical records and self-administered questionnaires.

Medical history, including age at diagnosis, detailed data regarding the tumor, and treatment and cumulative RAI dose received, were obtained from the patients' medical records. Follow-up duration was defined as the period between the date of diagnosis and the date of evaluation (in years).

We defined RAI therapy as the administration of RAI at an activity of 30 millicuries (mCi) or higher.

The patients filled out a questionnaire collecting information on obstetric details of the pregnancies, age at first pregnancy, number of children conceived, number of miscarriages, number of premature, stillborn, or malformed neonates, and whether the patient had visited a fertility clinic. Gynecological information collected included the following: menstrual history, menarche, use of current medication or contraceptive methods, surgery, and menopause or POI.

Menopause was defined as the reported cessation of spontaneous menses for 12 months without any other obvious pathological or physiological cause, whereas POI was defined as reported menopause at the age of 40 years or less.

The overall fertility rate was calculated by averaging all individual fertility rates, *i.e.*, the average number of children that a patient had at the end of her reproductive period.

### Hormonal data

Blood samples for measurement of AMH levels were obtained by venipuncture at any time during the menstrual cycle. Serum AMH levels were measured using electrochemiluminescence immunoassay (ECLIA; Elecsys AMH, Roche; detection limit 0.010 ng/mL [0.071 pmol/L], measuring range 0.01-23 ng/mL [0.071-164.2 pmol/L]). The reference values for AMH for ages 20-50 years, provided by the manufacturer, were as follows: 20-24 years, 1.66-9.49 ng/mL; 25-29 years, 1.18-9.16 ng/mL; 30-34 years, 0.67-7.55 ng/mL; 35-39 years, 0.78-5.24 ng/mL; 40-44 years, 0.10-2.96 ng/mL; 45-50 years, 0.05-2.06 ng/mL. For women with polycystic ovary syndrome (PCOS), the reference values were 2.41-17.10 ng/mL. For patients younger than 20 years, the reference values were 0.44-7.75 ng/mL for girls aged 12-14.9 years and 0.34-10.39 ng/mL (corresponding to 2.5th and 97.5th percentiles, respectively) for girls aged 15-18.9 years (10).

### Statistical analysis

The data collected were tabulated and statistically analyzed using the software Statistical Package for Social Sciences (SPSS; IBM Corp., Armonk, NY, USA). Numerical variables are expressed as mean ± standard deviation, whereas qualitative variables are expressed as numbers and percentages. Between-group comparisons of parametric data were performed using two-sided *t* test, while comparisons of nonparametric data were performed using the chi-square or Mann-Whitney *U* test. For all tests, a probability (p) below 0.05 was considered significant.

## RESULTS

In all, 64 participants between the ages of 16 and 51 years who were categorized as having a history of pregnancy (32/64) or no history of pregnancy (32/64) were eligible. Table 1 summarizes the patients' data, including age at diagnosis, type of malignancy, tumor size and characteristics, follow-up duration, and age at the last visit.

**Table 1:** Characteristics of patients and tumors

Characteristics		Total n = 64	History of pregnancy n = 32	No history of pregnancy n = 32	P values
Age at diagnosis (years)					
	Mean (SD)	14.1 (3.2)	14.3 (2.4)	13.9 (2.5)	0.018
	Median (min-max)	14.8 (4.5-18.5)	14.5 (4.5-18.3)	14.9 (4.7-18.5)	
Histopathology					
	Papillary thyroid carcinoma – n (%)	60 (94)	30 (94)	30 (94)	1.0
	Follicular thyroid carcinoma – n (%)	4 (6)	2 (6)	2 (6)	
Tumor size (cm)					
	Mean (SD)	2.8 (1.4)	2.3 (0.9)	3.2 (1.1)	0.014
	Median (min-max)	2.5 (0.7-7.0)	2.0 (0.7-4.8)	3.1 (1.3-7)	
Extrathyroidal extension – n (%)		41 (64)	21 (66)	20 (63)	0.79
Multifocality – n (%)		21 (33)	9 (28)	12 (38)	0.59
Lymph node metastasis – n (%)		44 (69)	21 (66)	23 (71)	0.59
Distant metastasis – n (%)		26 (41)	13 (41)	13 (41)	1.0
Follow-up duration (years)					
	Mean (SD)	14.9 (9.0)	18.1 (6.8)	11.6 (6.5)	0.004
	Median (min-max)	14.4 (1.1-34.7)	16.9 (2.84-34.7)	10.4 (1.1-29.8)	
Age at last visit (years)					
	Mean (SD)	28.9 (9.2)	32 (7.2)	25.5 (6.6)	0.003
	Median (min-max)	26.9 (16.5-51.6)	30.3 (18.5-51.6)	22.5 (16.5-40.4)	

The group with a history of pregnancy had smaller primary tumors (p = 0.014) and a higher mean age at diagnosis (*p* = 0.018) compared with the group without a history of pregnancy. Most patients in each group had papillary thyroid carcinoma (94%). Additionally, 26 (41%) DTC survivors had distant metastases, without significant difference between the groups.

Treatment details are shown in Table 2. All patients received RAI therapy. Significant differences were observed regarding age at the time of first RAI treatment (*p* = 0.018), with the group with a history of pregnancy being older than the group without a history of pregnancy. Differences between groups regarding menarche before RAI treatment were not statistically significant (*p* = 1).

**Table 2:** Treatment characteristics of the survivors included in the study

Characteristics		Total n = 64	History of pregnancy n = 32	No history of pregnancy n = 32	P values
Age at RAI treatment (years)					
	Mean (SD)	14.7 (3.2)	15.1 (2.5)	14.4 (2.7)	
	Median (min-max)	15.7 (5.4-18.9)	16.1 (8.0-18.9)	15.6 (5.4-18.9)	0.018
Menarche before RAI treatment – n (%)		51 (80)	26 (81)	25 (78)	1.0
Cumulative RAI activity (mCi)					
	Mean (SD)	250 (203)	261 (147)	239 (160)	0.68
	Median (min-max)	150 (30-1150)	165 (30-700)	150 (100-1150)	
Multiple RAI administrations – n (%)		22 (34)	14 (43)	8 (25)	0.09
Cumulative activity = 200 mCi – n (%)		20	13	7	0.17
Cumulative activity = 150 mCi – n (%)		25	16	9	0.12
AMH (n = 33)					
	AMH out of reference range	7 (10.9)	4 (12.5)	3 (9.3)	0.6
	AMH < 1 mg/mL – n (%)	8 (10.8)	6 (18.7)	2 (6.25)	0.2
	AMH < 2 SD – n (%)	10 (13.5)	6 (18.7)	4 (12.5)	0.6
AMH levels – median (min-max)		2.49 (0.01-7.81)	2.09 (0.01-6.63)	2.65 (0.01-7.36)	0.7

Abbreviations: AMH: anti-Müllerian hormone; mCi, millicurie; RAI, radioiodine; SD, standard deviation.

The group with a history of pregnancy received higher average RAI doses. However, no significant difference (*p* = 0.68) was observed regarding the cumulative RAI activity (mCi) among patients with *versus* without a history of pregnancy. In all, 22 patients were treated with more than one RAI dose. Among these patients, 14 had a history of pregnancy while 8 had no history of pregnancy, but this difference was not significant (p = 0.09). Additionally, no significant difference was observed between patients who received cumulative activity ≥ 200 mCi or ≥ 150 mCi (p = 0.17 and p = 0.12, respectively, relative to patients who received cumulative activities below these values). Serum AMH levels were measured at random moments, and results from 33 patients were available for analysis. As shown in Table 2, the median AMH values did not differ significantly between patients with and without a history of pregnancy. Eight patients had AMH levels below 1 mg/mL and they were equally distributed in both groups. Upon further analysis using standard deviations from the mean per age according to the manufacturer's datasheet, no difference was observed between both groups. Notably, AMH levels were more frequently out of the reference range in patients who received multiple RAI treatments (42.8%) compared with those who received a single RAI treatment (5.2%; p = 0.014).

Supplementary Table 1 shows the age distribution of the female survivors at the time of data collection. In all, 32 patients reported a total of 46 pregnancies, with 44 (95.6%) live births, one (2.2%) premature birth, and no stillborn or malformed neonates. The median age at first pregnancy was 24 years, and the fertility rate was 1.37. Notably, the current median age at first pregnancy in Brazil is approximately 21 years (11).

None of the study patients reported POI. Nine patients were 40 years or older at the time of the assessment, and two reported menopause, including one in the group with a history of pregnancy. Nine patients were using contraceptive methods at the time of the study, all of whom had a history of pregnancy. One patient reported surgical sterilization (hysterectomy) after a previous pregnancy.

Regarding gynecological manifestations, such as PCOS or endometriosis, 18.6% of the patients who had been pregnant reported these conditions compared with 6.2% of those without a history of pregnancy. A significant association between age at first pregnancy and the presence of these comorbidities (which could explain the difference between our study population and the general Brazilian population) was not observed.

A subanalysis was performed including only patients older than 21 years at their last visit, who had either been previously pregnant or were not using any contraceptive methods. The results of this subanalysis showed no significant differences in pregnancy rates concerning cumulative RAI activity, treatment regimen, or the time of the first RAI treatment between patients who became pregnant and those who did not.

The rates of gestational complications and malformations were very low, as shown in Supplementary Table 2.

## DISCUSSION

Our study analyzed the pregnancy rates in a cohort of 64 female CCSs who were treated with RAI and reached childbearing age. Half of these women had already experienced at least one pregnancy. Cumulative RAI activity – including multiple *versus* single treatment for the same cumulative activity of RAI – did not differ between groups with and without a history of pregnancy. Interestingly, the group without a history of pregnancy was younger at diagnosis and had larger tumors, but the aggressiveness of the disease was similar between the groups. Additionally, there was no evidence of the impact of RAI therapy for childhood DTC on adverse pregnancy outcomes, including miscarriage, premature birth, and stillbirth or congenital malformations, after a median follow-up period of 14.4 years when the study cohort was compared with women in the general population.

In this group of childhood DTC survivors, the median age at first pregnancy was 24 years, which is slightly higher than the corresponding median age in the Brazilian population, *i.e.*, currently approximately 21 years (12). However, some studies have found pregnancy delays among CCSs (13). The mean number of children per fertile woman in our study, adjusted for maternal age, was similar to that of the national average (1.37 *versus* 1.53, respectively) (14). Two previous studies in the Brazilian population found no increased risk of adverse pregnancy outcomes among young women with thyroid cancer. Rosário and cols. (15) evaluated 78 pregnant women who received low RAI activity and compared them to the general population, observing no increase in abortion and prematurity rates, birthweight, or offspring malformations. Brandão and cols. (16) evaluated abortion and fetal congenital anomaly rates in 66 pregnant women who received high RAI activity, confirming that it did not prevent pregnancy. Our data confirm the results of these studies in the Brazilian population. The studies mostly included women treated as young adults, while no consistent data exists in an exclusively pediatric population.

We found no increased miscarriage rate among DTC survivors (4.4%). Quenby and cols. observed a rate of spontaneous abortions in the normal population of 15.3% (17). Schlumberger and cols. (18) showed that the miscarriage rate among DTC survivors increased slightly after surgery but not after RAI therapy or treatment with high cumulative RAI activity. A French study (19) including a cohort of 45 patients with DTC diagnosed and treated before the age of 25 years found that the frequency of miscarriages was 17% among women who received > 3848 MBq (104 mCi) and 10% among those who received a cumulative RAI dose < 3848 MBq (104 mCi). These results are comparable with data from the general French population.

A meta-analysis of 22 articles conducted by Moon and cols. (6), including 5 case-control studies and 17 case series, found that the risk of miscarriage or abortion was increased in patients with DTC when compared with those without DTC. The odds of preterm labor and congenital anomalies were not significantly high. A subgroup analysis of patients with DTC categorized according to RAI treatment revealed that the risk of miscarriage, preterm labor, or congenital anomalies was not increased in patients treated with RAI compared with those who did not receive such treatment. In the present study, adverse pregnancy outcomes – such as stillbirths or congenital anomalies – were not reported.

Our study found that pregnancy rates were not affected by disease severity in patients with thyroid cancer who received RAI treatment. Interestingly, patients who did not become pregnant after RAI treatment had larger tumors than those who did become pregnant. This finding is aligned with results from a similar American study among women diagnosed with thyroid cancer during adolescence and young adulthood (20).

Additionally, our study found no significant effect of the cumulative RAI dose on reproductive characteristics and no significant differences between patients treated with multiple *versus* single RAI doses. Similar results were obtained in a subanalysis including only patients older than 21 years at their last visit. These results are consistent with findings from a US (20) and a recent Israeli (21) study including large female populations with thyroid cancer during adolescence and young adulthood; these studies revealed that RAI treatment – whether given alone or in multiple doses – did not affect fertility or pregnancy outcomes significantly. Similar results were also observed in a retrospective cohort study by Chow and cols. (22), who found no harmful effects on subsequent pregnancies in young women with DTC treated with high-dose RAI ablation, and a study conducted by Balenović and cols. (23), which found that a higher therapeutic dose (>100 mCi) did not significantly affect pregnancy outcomes.

Only a limited number of studies have been conducted on female survivors of childhood DTC. In fact, we only found three studies (8,9,23) that have examined this population. Aligned with our results, another retrospective study by Sarkar and cols. (24), including 20 women, found no overt evidence of genetic damage in children and adolescents. Additionally, no correlation was found between age at first therapy or total RAI dose and genetic damage. A study from Italy (9), including 72 women, found no association between fertility problems and RAI doses. Similarly, a retrospective cohort study (8) from the Netherlands on childhood DTC survivors reported no major reproductive abnormalities, which is consistent with our results.

Notably, we found in a previous study that female survivors of childhood DTC who underwent multiple RAI treatments did not experience a significant decrease in pregnancy rates, despite a decrease in serum AMH levels. This result was also observed in the present study, which found similar AMH levels between both groups, but this measurement was done randomly. Data on AMH level at the time of pregnancy in the group with a history of pregnancy were unavailable, and we recognize that the random measurements that we obtained could reflect a moment in which the ovarian reserve could have already decreased. These results are aligned with those from a recent meta-analysis by Piek and cols. (7), which also showed that RAI therapy for DTC is not associated with a long-term decrease in pregnancy rates. However, a significant decrease in AMH levels after RAI therapy is likely. The association between the decrease in AMH levels and its impact on pregnancy rates requires further clarification. Prospective studies aiming at determining, instead of pregnancy rates, the time between willingness to conceive and actual pregnancy could be potentially informative for future counseling for girls who are treated with RAI therapy.

A particular strength of our study was its population, which consisted solely of CCSs. The study included the largest cohort of pregnant female survivors of childhood DTC who underwent RAI treatment in Brazil. To ensure the validity of our results, we excluded women who had received other types of radiotherapy before pregnancy. Only women of childbearing age who had not used contraceptive methods were included in the study. However, as our study was retrospective in nature, some data may have been overlooked or biased, and the follow-up period of 14 years may not have been sufficient to detect late complications. Additionally, since many of the survivors were of childbearing age, the group without a history of pregnancy was younger, so their reproductive characteristics may still change in the long term. We used questionnaires to collect data, and as such, we did not have access to the Brazilian live birth registry. Last, the small sample size was a major issue and could have increased the chances of a statistical type II error; however, the study confirmed the findings of other studies that also found implications of RAI in pregnancy rates.

In conclusion, the results of the present study indicate no observational evidence suggesting important adverse effects of RAI on fertility or pregnancy outcomes among female survivors of childhood DTC. Prospective studies are needed to confirm these results and to compare them with a paired population without RAI treatment. We were unable to prove a direct impact of RAI on pregnancy rates. To verify a direct effect of RAI treatment on pregnancy, we need to follow prospectively the group of women without a history of pregnancy until they reach an age where they can no longer conceive or are confirmed to be infertile. Further studies including larger populations and longer follow-up periods are needed to clarify these issues properly.

## SUPPLEMENTARY MATERIAL

**Supplementary Table 1:** Age distribution of the female survivors at the time of data collection

	Total	History of pregnancy	No history of pregnancy
Distribution – n (%)	64 (100)	32 (100)	32 (100)
16-19 years	11 (17)	2 (6)	9 (28)
20-24 years	16 (25)	5 (15)	11 (34)
25-29 years	12 (18)	7 (22)	5 (15)
30-34 years	8 (13)	6 (19)	2 (7)
35-39 years	8 (13)	6 (19)	2 (7)
≥40 years	9 (14)	6 (19)	3 (9)

**Supplementary Table 2:** Characteristics of the group with a history of pregnancy

Characteristics	Pregnancy n = 32
Number of pregnancies – n	46
Live births – n (%)	44 (95.6)
Miscarriage – n (%)	2 (4.4)
Premature birth – n (%)	1 (2.2)
Stillborn or malformed neonates – n (%)	0
Menopause – n (%)	1 (3.1)
Premature ovarian insufficiency – n (%)	0
Hysterectomy – n (%)	1 (3.1)
Use of contraceptives – n (%)	9 (28.1)
Gynecological changes (PCOS, endometriosis) – n (%)	6 (18.6)

Abbreviation: PCOS, polycystic ovarian syndrome.
